# Colonic Ganglioneuroma: A Combined Single-Institution Experience and Review of the Literature of Forty-Three Patients

**DOI:** 10.3390/diseases11020069

**Published:** 2023-05-06

**Authors:** Hisham F. Bahmad, Sally Trinh, Linda Qian, Kristy Terp, Ferial Alloush, Mohamad K. Elajami, Ekim Kilinc, Robert Poppiti

**Affiliations:** 1 Department of Pathology and Laboratory Medicine, Mount Sinai Medical Center, Miami Beach, FL 33140, USA; ferial.alloush@msmc.com (F.A.); ekim.kilinc@msmc.com (E.K.); robert.poppiti@msmc.com (R.P.); 2 Herbert Wertheim College of Medicine, Florida International University, Miami, FL 33199, USA; strin007@med.fiu.edu (S.T.); lqian005@med.fiu.edu (L.Q.); kterp001@med.fiu.edu (K.T.); 3 Department of Internal Medicine, Mount Sinai Medical Center, Miami Beach, FL 33140, USA; mohamad.elajami@msmc.com; 4 Department of Pathology, Herbert Wertheim College of Medicine, Florida International University, Miami, FL 33199, USA

**Keywords:** ganglioneuroma, colon, case series, diverticulosis

## Abstract

Ganglioneuromas (GNs) are rare, benign tumors composed of ganglion cells, nerve fibers, and glial cells. Three types of colonic GN lesions exist: polypoid GNs, ganglioneuromatous polyposis, and diffuse ganglioneuromatosis. Less than 100 cases of GN are documented in the literature. A 10-year retrospective search of the pathology database at our institution identified eight cases of colonic GNs. All cases were incidental. Seven of the eight cases presented with colonoscopy findings of small sessile polyps (ranging between 0.1 and 0.7 cm) treated with polypectomy, whereas one case showed a 4 cm partially circumferential and partially obstructing mass in the ascending colon, treated with right hemicolectomy. Almost two-thirds of the cases (5/8) demonstrated associated diverticulosis. All cases were positive for S100 protein and Synaptophysin via immunohistochemistry (IHC). No syndromic association was identified in any of the cases. We also conducted a comprehensive review using PubMed to identify cases of colonic GN reported in the literature. In total, 173 studies were retrieved, among which 36 articles met our inclusion criteria (35 patients and 3 cases on animals). We conclude that while most GNs are incidental and solitary small sessile lesions, many can be diffuse and associated with syndromes. In these cases, the tumor can result in bowel obstruction simulating adenocarcinoma.

## 1. Introduction

Ganglioneuromas (GNs) are rare, benign tumors of undifferentiated neural crest cells of the autonomic nervous system, specifically the sympathetic nervous system. They belong to a group of tumors known as peripheral neuroblastic tumors (PNTs) that include neuroblastomas, ganglioneuroblastomas, and GNs. PNTs tend to arise in the adrenal gland and retroperitoneal ganglia; hence, the colonic location of GN is unusual [1]. GNs are derived from neural crest cells that are embryonic, in their final stage of maturation, with their location being anywhere on the sympathetic chain [2].

PNTs arise from the neural crest cells that migrate to form the sympathetic chain ganglia and adrenal medulla [3]. They can develop at any point during the migration and differentiation process of these cells, resulting in a diverse spectrum of tumors with varying clinical behavior. Various genetic mutations and chromosomal abnormalities have been implicated in the development of PNTs, including *MYCN* amplification, deletions of 1p and 11q, and gain of 17q [1,4].

GNs are composed of ganglion cells, nerve fibers, and glial cells [5]. Proliferations of GNs in the gastrointestinal tract are rare, with most being found in the colon. Three types of colonic GN lesions exist: polypoid GNs (PG), ganglioneuromatous polyposis (GP), and diffuse ganglioneuromatosis (DG) [6,7]. Grossly, GNs can be either sessile (without a stalk) or pedunculated (with a stalk). GP has several similarly sized polyps identical to PGs and may also be sessile or pedunculated grossly and mucosal or submucosal microscopically. DGs are normally larger in size, growing up to 17 cm. These lesions are poorly demarcated, nodular, and involve the myenteric plexus. They may be either mucosal or transmural. Mucosal lesions of the DGs are found more in adults, while mucosal and submucosal may be seen in children [6]. Patients are usually asymptomatic, and GNs are normally detected during screening colonoscopies but may have symptoms, including abdominal pain, bleeding, constipation, weight loss, or obstruction. These symptoms depend on the polyp size and location [8].

**Figure 1:**
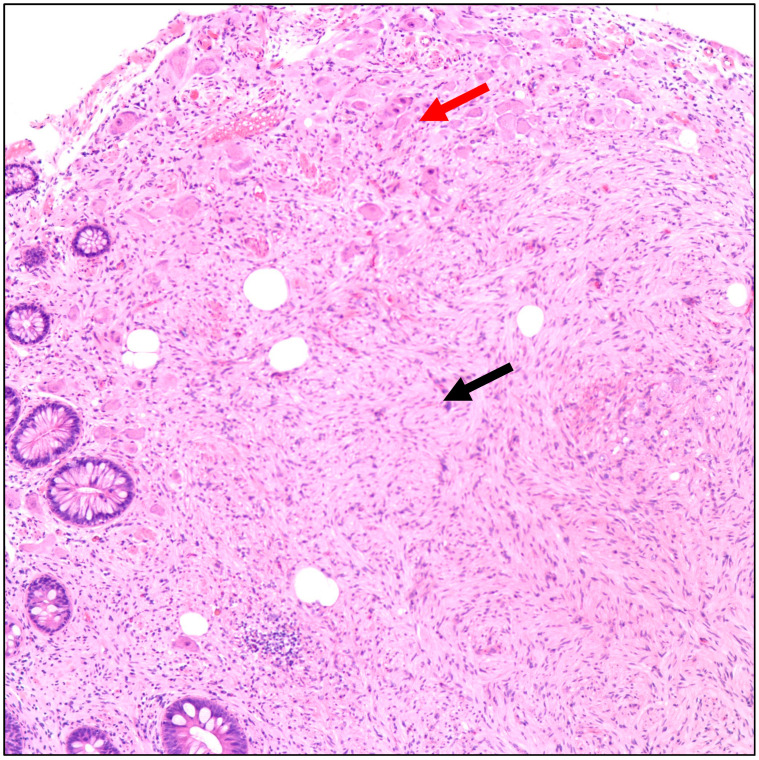
Hematoxylin and eosin (H&E) microscopic image of a ganglioneuroma. The image shows proliferation of spindle cells with wavy nuclei (Schwann cells, black arrow) and scattered large cells with eccentric vesicular nuclei, prominent nucleoli, and abundant amphophilic cytoplasm (Ganglion cells, red arrow) (100× magnification).

**Figure 2:**
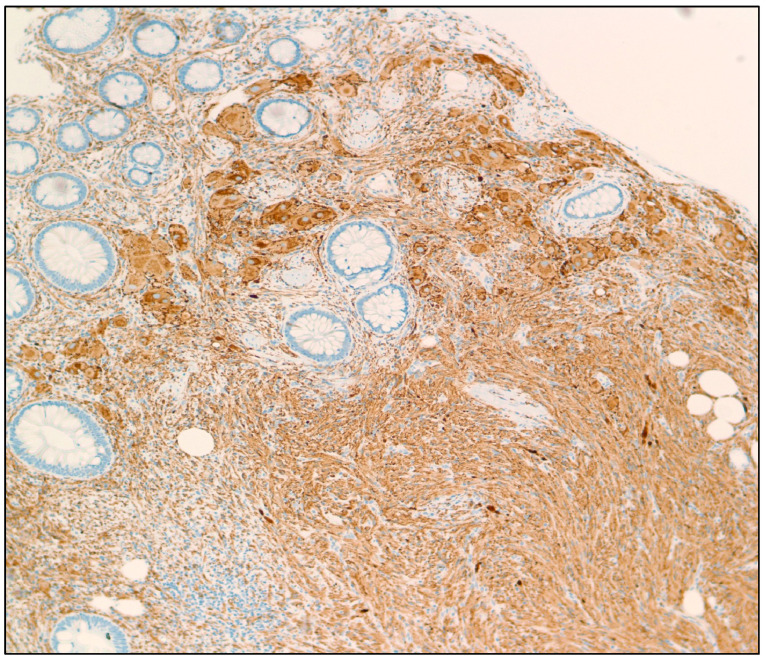
Synaptophysin IHC stain of a GN showing cytoplasmic immunoreactivity in tumor cells (100× magnification).

**Figure 3:**
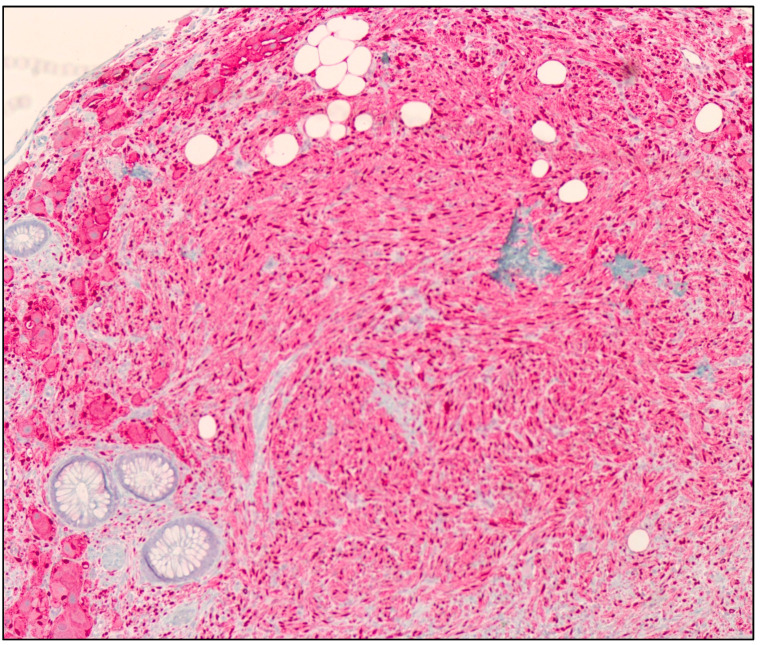
S100 protein IHC stain of a GN showing cytoplasmic and nuclear immunoreactivity in tumor cells (100× magnification).

Due to their rarity, the incidence of GNs is not exactly known. Based on our recent literature review, we suspect that there are less than 100 documented cases of colonic GN, and less than 5 cases in animals with colonic GNs. Immunohistochemistry (IHC) is often used as a method to detect the specific protein markers, which help with the diagnosis of colonic GNs. Histologically, GNs reveal mature ganglion cells that are large with axons, Schwann cells, fibrous stroma, and satellite cells (Figure 1). IHC of these lesions may display positivity with neurofilaments, synaptophysin (Figure 2), chromogranin, S100 protein (Figure 3), glial fibrillary acidic protein (GFAP), and CD56 in ganglion and Schwann cells [9]. To the best of our knowledge, our study presents the biggest published case series and review of the literature conducted to date.

The diffuse form of GNs is strongly associated with multiple syndromes, including Neurofibromatosis 1 (NF1), also known as von Recklinghausen’s disease, Multiple Endocrine Neoplasia type 2B (MEN2B), and Cowden Syndrome. In many cases, GNs are the first sign of these syndromes [10]. In MEN2B, there is a gain of function in the *RET* proto-oncogene on chromosome 10, resulting in increased growth and differentiation signals in different tissues, including those of neural crest origin, ultimately resulting in the formation of GNs [11]. A single 918 Met to Thr mutation in exon 16 is responsible for over 95% of cases of MEN2B and is specific for this disorder [12]. In Cowden Syndrome, a mutation in the *PTEN* gene results in a frameshift that changes glutamic acid at codon 91 to phenylalanine and a premature stop codon at position 4. This causes loss of function from truncation of a phosphatase protein or mRNA decay, which negatively regulates cell cycle progression, cell proliferation, and apoptosis pathways [13]. Loss of this critical function contributes to oncogenesis that may lead to the growth of GNs. In NF1, the *NF1* gene is mutated on chromosome 17, which encodes neurofibromin, a tumor suppressor protein found in the nervous system that prevents cell overgrowth of neurons, microglia, oligodendrocytes, Schwann cells, and astrocytes. As a result, GNs may occur from proliferation of these cells [14].

There are various managements for GNs, depending on the clinical history and location or size of polyps. Polypectomy is one option that may be curative for certain subgroups. Another would be either partial or total colectomy, depending on polyp location. It is also imperative to test for associated syndromes or systemic disease when the suspicion of GN-associated syndromes is high, such as calcitonin levels for MEN2B [15]. Perhaps due to the rarity of colonic GNs and their non-malignant character, the published literature on these tumors is comprised primarily of case reports and small retrospective studies. Studying additional cases of colonic GNs may serve to better characterize the patients who are likely to develop these tumors.

The objectives of our study include collecting the clinical and pathological data of patients with a diagnosis of GN of the colon treated at our institution during the past decade and describing the clinical presentation and imaging results. We also conducted a comprehensive review using PubMed to identify cases of colonic GN reported in world literature.

## 2. Methods

### 2.1. Study Design, Setting, and Objectives

The pathological database of surgical specimens from patients with GN of the colon during a ten-year period, from 1 January 2013 to 15 March 2023, at Mount Sinai Medical Center (Miami Beach, FL, USA), was reviewed.

### 2.2. Ethical Considerations

Approval of the Institutional Review Board of Mount Sinai Medical Center of Florida was granted prior to commencement of the study (protocol code FWA00000176; 7 March 2023). All protocols followed in our retrospective cohort study were performed in accordance with guidelines and regulations of The Code of Ethics of the World Medical Association (Declaration of Helsinki). The study was conducted in a manner that ensures confidentiality of patients. Informed consents from patients were waived due to the retrospective nature of our study.

### 2.3. Patients’ Selection and Clinicopathological Parameters of Patients

Inclusion criteria included patients who underwent colonoscopy or other colon sampling procedures during the specified time period. Only patients having a diagnosis of colonic GN confirmed by a pathological examination after coloscopy or other colon sampling procedures were included. Regular screening that begins at age 45 is performed to prevent colorectal cancer and find it early, as recommended by the U.S. Preventive Services Task Force (adults aged 45 to 75 years are screened for colorectal cancer). No exclusion criteria were present. Clinical and pathological parameters of patients were retrospectively retrieved from electronic medical records, including age, clinical data (signs and symptoms, medical history, imaging results), site, type of surgery, indication of surgery, pathological diagnosis, and histopathologic characteristics.

### 2.4. Review of Literaure

We conducted a comprehensive review and performed a thorough search using PubMed as the primary database, for mesh terms, keywords, and combinations as follows: “colon” and “ganglioneuroma”. All published articles from inception to 15 March 2023, were included. Eligibility criteria for article selection included articles tackling GNs of the colon specifically. There were 137 articles excluded since they did not meet our inclusion criteria. Non-English language was an exclusion criterion. It was interesting to find three articles reporting cases of colonic GN in animals, so we included those articles in our analysis. Of note, the review of the literature also included patients in our case series.

## 3. Results

### 3.1. Mount Sinai Medical Center of Florida Experience

**Figure 4:**
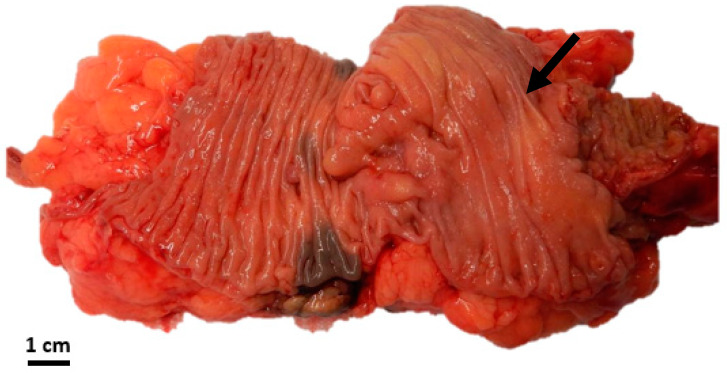
Gross image of the largest tumor showing the ill-defined nature of the mass (black arrow).

A comprehensive pathology database review spanning the last ten years at our institution revealed GNs identified in eight patients (Table 1). The median age at the time of diagnosis was 57 years, ranging from 51 to 78 years. All cases were discovered incidentally during routine screening coloscopy. The male-to-female ratio was 5:3. Histopathologic features are summarized in Table 1, with the mean size of GN being 0.8 ± 1.3 cm. Interestingly, one case showed a 4 cm partially circumferential and partially obstructing mass in the ascending colon, treated with right hemicolectomy (Figure 4). Almost two-thirds of the cases (5/8) demonstrated associated diverticulosis (Table 1). All cases were positive for S100 protein and Synaptophysin via IHC. No syndromic association was identified in any of the cases. Upon follow-up, none of the patients had significant findings in their colonoscopies (when performed). Further, no further specific treatment was given to any of the patients as regards their colonic GN.

### 3.2. Comprehensive Review of Literature

In our study, a total of 173 studies were retrieved using this search algorithm. All studies were assessed for eligibility, after which 36 articles (35 patients and 3 cases on animals) were included based on their abstracts and deemed eligible for whole-text analysis. Additionally, GNs appear not to be frequently reported in the pediatric population. Combining our eight cases with the 35 patients reported in the literature, we summarize the characteristics of 43 patients in Table 2. The mean age of patients was 50.1 ± 18.5 years (median 51 years and range 5–81 years). The male-to-female ratio was 2.58:1. Among the 43 patients, 55.8% had PGs, 32.6% had DG, and 11.6% had GP. No associated pathology was seen in three-fourths of patients (74.5%), while 11.6% of patients had associated diverticulosis, and 9.3% had adenocarcinomas. The mean size of GN was 2.5 cm, with a wide standard deviation of 3.6 cm. In two-thirds of patients, polypectomy was performed while the rest underwent colectomies (Table 2).

Common locations for colonic GNs were the ascending and sigmoid colons, although a large number of cases were also reported to be diffuse throughout the entire colon, particularly the cases of diffuse ganglioneuromatosis (DG) and ganglioneuromatous polyposis (GP). The majority of these cases were not incidental. Common associated symptoms were abdominal pain and fecal occult blood loss. A majority of the cases did not have associated syndromes. Associations included NF1, polyposis coli, Cowden syndrome, familial hamartomatous polyposis syndrome, PTEN hamartoma tumor syndrome, familial adenomatous polyposis syndrome, and syndrome of watery diarrhea, hypokalemia, and achlorhydria. Common histological features of the colonic GNs included the proliferation of ganglion cells and spindle cells, and in many cases, the IHC stains were S100 protein positive. Management included polypectomy or colectomy. One case reported a 43-year-old man with intermittent lower abdominal pain for 6 weeks with nausea and a 2-day history of diarrhea who had polypectomy of a polypoid solitary GN with a hot snare during colonoscopy that resulted in symptom resolution [16]. In another case, a 67-year-old man with diffuse polyposis and associated NF1 presented with lower abdominal pain that resolved after total colectomy and proctectomy [17] (Table 3).

There were three articles referring to animals, including a dog, horse, and steer [18,19,20]. In the case of the horse, the authors described an 8-year-old mixed-breed horse with GN in a 25 cm segment of the small colon [19]. An interesting finding was described in the case of the colonic GN of a dog, where a 5-month-old female Great Dane puppy was suffering from hematochezia, tenesmus, and rectal prolapse. As such, 10 cm long segments of the colon and rectum were resected, showing diffuse mucosal and submucosal thickening with multiple polypoid nodules on gross examination. Histologically, the colorectal hamartomatous polyps revealed ganglioneuromatosis. Quantitative multiplex polymerase chain reaction testing was performed revealing the duplication of *PTEN*. This observation, where hamartomatous colorectal lesions with *PTEN* mutation occur, is similar to human Cowden syndrome [18]. All animal case reports displayed diffuse ganglioneuromatosis. One of the cases was noted to be S100 protein positive. Management included colectomy without specific follow-up (Table 4).

**Table 3:** Summary of 35 cases reported on humans in literature.

Ref.	Year	Age (Years)	Sex	Location in Colon	Category	Associated Symptoms	Associated Syndromes	Size	Associated Pathologies	IHC Stains	Management
[21]	2022	38	F	Sigmoid colon	DG	-	-	30 mm	Adenocarcinoma	S100 protein (+)	Colectomy
[22]	2021	73	M	Splenic flexure	PG	-	-	10 mm	-	-	Polypectomy
[16]	2021	44	M	Ascending colon and hepatic flexure	GP	Abdominal pain	-	-	Adenocarcinoma	Chromogranin, synaptophysin, CD56, and CK20 (+)	Colectomy
		43	M	Ascending colon	PG	Abdominal pain	-	8 mm	-	-	Polypectomy
[10]	2021	9	M	Cecum	DG	Abdominal pain	-	5 cm	-	GFAP and S100 protein (+)	Polypectomy
[6]	2020	84	M	Ascending and descending colons	PG	Hematochezia	-	3 to 4 mm range	-	-	Polypectomy
[23]	2020	40	M	Ascending colon	PG	Positive fecal occult blood	-	15 mm	-	S100 protein and synaptophysin (+)	Polypectomy
[24]	2020	68	M	Sigmoid colon	PG	Positive fecal occult blood	-	11 mm	Mucosal neurofibromas	S100 protein (+)	Colectomy
[25]	2018	50	M	Splenic flexure	PG	-	-	7 mm	-	S100 protein and synaptophysin (+)	Polypectomy
[5]	2017	65	M	Ascending colon	PG	-	-	6 mm	-	S100 protein (+)	Polypectomy
[17]	2017	67	M	Colon (diffuse)	DG	Abdominal pain	NF1	-	-	S100 protein and synaptophysin (+)	Right hemicolectomy
[26]	2017	43	M	Colon (diffuse)	PG	Hematochezia	PHTS	1 to 6 mm range	-	-	Polypectomy
[8]	2016	51	F	Descending colon	PG	-	-	10 mm	-	S100 protein and NSE (+)	Polypectomy
[27]	2016	60	M	Colon (diffuse)	GP	-	Polyposis coli and Cowden	0.3 to 2.3 cm range	-	S100 protein (+)	Total colectomy
[28]	2016	51	F	Colon (diffuse)	PG	-	-	2 to 5 mm range	-	S100 protein and NSE (+)	Polypectomy
[15]	2015	70	M	Rectosigmoid colon	DG	Diarrhea	-	8.5 cm	-	S100 protein and chromogranin (+)	Colectomy
		35	M	Ascending colon	DG	-	-	5.5 cm	-	S100 protein, chromogranin, and CD56 (+)	Right hemicolectomy
[29]	2015	38	M	Colon (diffuse)	DG	Diarrhea and weight loss	HPS	3 to 5 mm range	-	S100 protein (+)	Polypectomy
[30]	2015	43	M	Cecum	PG	Hematochezia	-	6 mm	-	S100 protein (+)	Polypectomy
[31]	2015	71	M	Colon (diffuse)	DG	Positive fecal occult blood	-	-	-	S100 protein, CD56, NSE, and Neurofilament protein (+)	Polypectomy
[32]	2015	54	M	Colon (diffuse)	DG	Hematochezia and abdominal pain	-	0.1 to 8 cm range	-	S100 protein and Neurofilament protein (+)	Polypectomy
[33]	2013	68	F	Descending colon	PG	-	-	16 cm	-	S100 protein and NSE (+)	Colectomy
[34]	2012	57	M	Colon (diffuse)	DG	Hematochezia	-	7 mm	-	S100 protein, synaptophysin, and chromogranin (+)	Polypectomy
[35]	2013	7	M	Colon (diffuse)	DG	Abdominal pain	-	-	-	S100 protein (+)	Colectomy
[36]	2012	61	M	Descending colon	PG	-	-	6 mm	-	S100 protein (+)	Polypectomy
[37]	2012	42	M	Colon (diffuse)	DG	Hematochezia	Cowden	-	Adenocarcinoma	-	Colectomy
[38]	2009	38	F	Transverse and sigmoid colon	DG	Abdominal pain	-	6 to 7 cm range	Adenocarcinoma	S100 protein (+)	Colectomy
[39]	2009	14	F	Colon (diffuse)	DG	Diarrhea	WDHA	-	-	-	Colectomy
[40]	2009	5	F	Sigmoid colon	PG	Abdominal pain	-	-	-	-	Colectomy
[41]	2008	48	F	Sigmoid colon	PG	-	-	-	Diffuse melanosis coli	S100 protein and NSE (+)	-
[42]	2006	41	M	Colon (diffuse)	GP	Hematochezia	-	1 to 2 mm range	-	S100 protein (+)	Polypectomy
[43]	2005	29	M	Colon (diffuse)	GP	Diarrhea	-	5 to 15 mm range	-	S100 protein (+)	Polypectomy
[44]	2006	42	M	Cecum	DG	Constipation	-	10 cm	-	S100 protein (+)	Ileocolectomy
[45]	1999	77	F	Cecum	GP	Abdominal pain	-	3 to 4 cm range	-	S100 protein and NSE (+)	Polypectomy
[46]	1998	54	M	Hepatic flexure	PG	-	-	5 mm	-	S100 protein (+)	-

Abbreviations: DG: Diffuse ganglioneuromatosis; F: female; GFAP: Glial Fibrillary Acidic Protein; GP: Ganglioneuromatous polyposis; HPS: Hamartomatous Polyposis Syndrome; IHC: Immunohistochemistry; M: male; NF1: Neurofibromatosis type 1; NSE: Neuron-Specific Enolase; PG: Polypoid ganglioneuroma; PHTS: PTEN hamartoma tumor syndrome; Ref.: Reference; WDHA: Syndrome of Watery Diarrhea, Hypokalemia, and Achlorhydri.

**Table 4:** Summary of 3 cases reported on animals in literature.

Ref.	Year	Species	Age	Sex	Location in Colon	Category	Size	IHC Stains	Management
[20]	1990	Steer	7 months old	-	Descending colon	DG	-	NSE and Neurofilament protein (+)	Colectomy
[19]	2007	Horse	8 years old	-	Colon (40 cm from anus)	DG	3 to 15 mm range	S100 protein and GFAP (+)	-
[18]	2011	Dog	5 months old	F	Colon and rectum	DG	2 to 8 mm range	-	Colectomy

Abbreviations: DG: Diffuse ganglioneuromatosis; F: female; GFAP: Glial Fibrillary Acidic Protein; IHC: Immunohistochemistry; NSE: Neuron-Specific Enolase; Ref.: Reference.

## 4. Discussion

In our study, we combined eight cases of colonic GNs diagnosed at our institution with 35 patients reported in the literature (43 patients in total). The mean age at presentation was 50.1 years. GNs are more commonly diagnosed in men than women, with the majority of cases located in the ascending and sigmoid colons and a good number of cases presenting with diffuse GNs throughout the colon. No associated pathology was seen in three-fourths of patients, while 11.6% of patients had associated diverticulosis. Nevertheless, it is estimated that around 15% of individuals will have diverticulosis by the age of 50 during routine-screening colonoscopy [47,48]. Therefore, we anticipate that this might be an incidental finding. In around 65.1% of patients, polypectomy was performed, while the rest underwent colectomies (Table 2).

Several studies have investigated the molecular genetics of GNs, with a particular focus on the involvement of the *RET* proto-oncogene and its downstream signaling pathways, such as AKT/mTOR activation [49]. Mutations in *RET* have been identified in a subset of GNs and are thought to disrupt the normal development and maintenance of the enteric nervous system [50,51]. Other genes implicated in GN development include *NF1*, *SDHB*, and *SDHD*, which have been associated with hereditary NF and paraganglioma syndromes [52,53]. Additionally, a study referred to the role of ERBB3 as a marker of a ganglioneuroblastoma/ganglioneuroma-like expression in neuroblastic tumors [54].

The limitations of this literature review included language barriers, as many articles were in languages other than English (Japanese, Chinese, French, and Danish) and, hence, were excluded.

The differential diagnosis of colonic GNs includes other benign tumors, such as neurofibromas, schwannomas, lipomas, and leiomyomas. These tumors are among the most common benign neoplasms affecting the soft tissues, and understanding their pathogenesis and genetic background is essential for accurate diagnosis and management. The accurate diagnosis of GNs requires careful histopathological examination, with characteristic features, including the presence of mature ganglion cells, spindle-shaped cells, and collagenous stroma.

In conclusion, although GNs, neurofibromas, schwannomas, leiomyomas, and lipomas are all benign tumors, each has unique histopathological features that can help distinguish them from one another. A thorough understanding of these features is essential for accurate diagnosis and management of these tumors.

## 5. Conclusions

While most GNs are incidental and solitary small sessile lesions, many can be diffuse and associated with syndromes. In these cases, the tumor can result in bowel obstruction simulating adenocarcinoma.

## Figures and Tables

**Table 1:** Clinicopathological characteristics of patients.

Clinicopathological Variables	Number of Patients
Mode of presentation	
Non-incidental	0
Incidental during routine screening colonoscopy	8
Sex	
Male	5
Female	3
Location	
Cecum	1
Ascending colon	2
Transverse colon	2
Descending colon	1
Sigmoid colon	2
Type	
PG	8
GP	0
DG	0
Associated diverticulosis	
No	3
Yes	5
Management	
Polypectomy	7
Colectomy	1

**Table 2:** Clinicopathological characteristics of 43 patients combining our cases with the cases reported in literature.

Clinicopathological Variables	Number of Patients (%)
Sex	
Male	31 (72.1%)
Female	12 (27.9%)
Location	
Ascending and descending colons	1 (2.3%)
Ascending colon	7 (16.3%)
Cecum	5 (11.6%)
Hepatic flexure	1 (2.3%)
Transverse colon	2 (4.7%)
Splenic flexure	2 (4.7%)
Descending colon	4 (9.3%)
Sigmoid colon	6 (14.0%)
Transverse and sigmoid colon	1 (2.3%)
Rectosigmoid colon	1 (2.3%)
Colon (diffuse)	13 (30.2%)
Category	
PG	24 (55.8%)
DG	14 (32.6%)
GP	5 (11.6%)
Associated pathologies	
None	32 (74.5%)
Diverticulosis	5 (11.6%)
Diffuse melanosis coli	1 (2.3%)
Adenocarcinoma	4 (9.3%)
Mucosal neurofibromas	1 (2.3%)
Management	
Polypectomy	28 (65.1%)
Colectomy	15 (34.9%)

Abbreviations: DG: Diffuse ganglioneuromatosis; F: female; GP: Ganglioneuromatous polyposis; M: male; PG: Polypoid ganglioneuroma.

## Data Availability

Not applicable.
